# Use of Machine Learning to Investigate the Quantitative Checklist for Autism in Toddlers (Q-CHAT) towards Early Autism Screening

**DOI:** 10.3390/diagnostics11030574

**Published:** 2021-03-22

**Authors:** Gennaro Tartarisco, Giovanni Cicceri, Davide Di Pietro, Elisa Leonardi, Stefania Aiello, Flavia Marino, Flavia Chiarotti, Antonella Gagliano, Giuseppe Maurizio Arduino, Fabio Apicella, Filippo Muratori, Dario Bruneo, Carrie Allison, Simon Baron Cohen, David Vagni, Giovanni Pioggia, Liliana Ruta

**Affiliations:** 1 National Research Council of Italy (CNR)—Institute for Biomedical Research and Innovation (IRIB), 98164 Messina, Italy; elisa.leonardi@istitutomarino.it (E.L.); stefania.aiello@istitutomarino.it (S.A.); flavia.marino@irib.cnr.it (F.M.); david.vagni@cnr.it (D.V.); giovanni.pioggia@cnr.it (G.P.); liliana.ruta@cnr.it (L.R.); 2 Department of Engineering, University of Messina, 98166 Messina, Italy; gcicceri@unime.it (G.C.); davidedipietro92@hotmail.it (D.D.P.); dbruneo@unime.it (D.B.); 3 Center for Behavioral Sciences and Mental Health, National Institute of Health, 00161 Rome, Italy; flavia.chiarotti@iss.it; 4 Child and Adolescent Neuropsychiatry Unit, Department of Biomedical Sciences, University of Cagliari and “G. Brotzu” Hospital Trust, 09124 Cagliari, Italy; antonellagagliano.npi@gmail.com; 5 Centro Autismo e Sindrome di Asperger ASLCN1, 12084 Mondovì, Italy; giuseppe.arduino@aslcn1.it; 6 IRCCS Stella Maris Foundation, Calambrone, 56128 Pisa, Italy; fabio.apicella@fsm.unipi.it (F.A.); filippo.muratori@fsm.unipi.it (F.M.); 7 Department of Clinical and Experimental Medicine, University of Pisa, 56126 Pisa, Italy; 8 Autism Research Centre, Department of Psychiatry, University of Cambridge, Cambridge CB2 8AH, UK; cla29@cam.ac.uk (C.A.); sb205@cam.ac.uk (S.B.C.)

**Keywords:** Q-CHAT, early screening, machine learning, autism

## Abstract

In the past two decades, several screening instruments were developed to detect toddlers who may be autistic both in clinical and unselected samples. Among others, the Quantitative CHecklist for Autism in Toddlers (Q-CHAT) is a quantitative and normally distributed measure of autistic traits that demonstrates good psychometric properties in different settings and cultures. Recently, machine learning (ML) has been applied to behavioral science to improve the classification performance of autism screening and diagnostic tools, but mainly in children, adolescents, and adults. In this study, we used ML to investigate the accuracy and reliability of the Q-CHAT in discriminating young autistic children from those without. Five different ML algorithms (random forest (RF), naïve Bayes (NB), support vector machine (SVM), logistic regression (LR), and K-nearest neighbors (KNN)) were applied to investigate the complete set of Q-CHAT items. Our results showed that ML achieved an overall accuracy of 90%, and the SVM was the most effective, being able to classify autism with 95% accuracy. Furthermore, using the SVM–recursive feature elimination (RFE) approach, we selected a subset of 14 items ensuring 91% accuracy, while 83% accuracy was obtained from the 3 best discriminating items in common to ours and the previously reported Q-CHAT-10. This evidence confirms the high performance and cross-cultural validity of the Q-CHAT, and supports the application of ML to create shorter and faster versions of the instrument, maintaining high classification accuracy, to be used as a quick, easy, and high-performance tool in primary-care settings.

## 1. Introduction

Autism is a set of neurodevelopmental conditions characterized by impairments in social communication with repetitive, restricted interests and behaviors, and atypical reactivity to sensory stimuli [1]. Autism is a lifelong condition in which the severity and intensity of symptoms are heterogeneous, and first signs occur in early childhood with different developmental trajectories [2]. Early screening and developmental surveillance represented a primary goal in the past two decades, and many screening tools were developed and tested. However, the performance, classification accuracy, and reliability of those screening instruments vary depending on different settings, samples, and screening designs, thereby posing critical issues for clinical application [3,4,5,6,7]. Among the most popular and replicated screening tools, the Modified Checklist for Autism in Toddlers (M-CHAT) and the subsequent revised M-CHAT/ Revised with Follow-Up (RF) [8] were applied in large mixed samples including both high- and low-likelihood groups, demonstrating low-to-moderate accuracy in detecting autism [9]. Other screening tools, such as the Social Communication Questionnaire (SCQ), reported poor balance between sensitivity and specificity in high-likelihood toddlers [10], while measures such as the Screening Tool for Autism in Two-year-olds (STAT) [11] and the Baby and Infant Screen for Children with aUtIsm Traits (BISCUIT) [12] were tested only in case–control studies, requiring further prospective population studies. With the shift from a categorical to a dimensional approach to autism diagnosis, a quantitative measure of autistic traits, the Quantitative CHecklist for Autism in Toddlers (Q-CHAT) was tested in different sample populations and cultures, in both case–control studies and primary-care settings, displaying fair-to-good psychometric properties and predictive validity, and good cultural stability [13,14,15,16,17,18,19]. A short Q-CHAT version, the Q-CHAT-10, including the 10 best predictive items was also developed [20], aiming to create a quick marker tool suitable for the time constraints of pediatric check-ups and to help further reduce the delay for potential referrals.

Most recently, computational intelligence and machine learning (ML) were applied to behavioral science, and provided novel opportunities to improve predictive accuracy and classification reliability in relation to early screening, detection, and autism diagnosis. ML algorithms are able to support autism screening and diagnosis by improving the sensitivity and specificity of the screening and diagnostic tools, and by helping to identify the least number of items maintaining satisfying classification accuracy. Classification accuracy is the number of correct predictions from all produced predictions, multiplied by 100 to turn it into a percentage. Classification accuracy of 0.50 indicates a random prediction of an independent variable, while accuracy > 0.90 indicates excellent predictive validity. One of the first studies related to the use of ML to diagnostic tools was conducted by Wall et al. (2012) [21], who applied ML algorithms to the Autism Diagnostic Observation Schedule (ADOS, Module 1). Eight items were able to classify autism with nearly 100% sensitivity and 94% specificity. The 8-question ML model published by Wall was replicated in two independent datasets by Duda and colleagues (2014) [22], and by Bone and colleagues [23], finding performance (measured as balanced accuracy) to be 90.2% and 94% against the best estimated clinical diagnosis, respectively. Subsequently, sparsifying ML models were applied to ADOS Modules 2 and 3 (for autistic children with verbal communication) finding classification accuracy of 93% for Module 2 and 95% for Module 3, selecting the 10 best items [24]. Results were validated in an independent study by Kosmicki and colleagues (2015) [25], who found that 9 and 12 items, respectively, from the ADOS (Modules 2 and 3) were able to detect autism spectrum disorder (ASD) risk with accuracy of 97.71% and 97.66%, respectively. In another study, ML was used to classify autism versus attention deficit hyperactivity disorder (ADHD) from Social Responsiveness Scale (SRS) codes [26]. Accuracy of 96.5% from only five items was achieved [27]. These findings were replicated in a larger dataset, using six items of the SRS, in a recent study by Washington and colleagues (2020) [28]. Furthermore, Bone and colleagues (2016) [29] applied ML strategies combining codes from both the SRS [26] and the Autism Diagnostic Interview-Revised (ADI-R) [30]. Processing items from multiple instruments, the ML algorithm was able, using only five behavioral codes, to detect autism with 89.2% sensitivity and 59.0% specificity. Subsequent studies using ML for improving the autism diagnosis (and screening) process showed performance in line with the previous results, supporting the hypothesis that ML is an effective way to build objective, quantitative models with few features to distinguish children with autism from children outside of the autism spectrum [31,32]. Very recently, ML was applied to datasets collected using a mobile application called AutismTests [33]. The AutismTests app was developed to screen for autism in children, adolescents, and adults using the short forms of the Autism Spectrum Quotient (AQ-10) and the Q-CHAT (Q-CHAT-10), respectively. In the first study, Thabath and colleagues (2019) [34] analyzed the adult version of the AQ-10 using new rule-based machine learning (RML) and were able to achieve about 90% accuracy, 87% sensitivity, and about 90% specificity. In the second study, the same authors applied the naïve Bayes algorithm to the AQ-10 and found similar accuracy of 92.8%, 91.3%, and 95.7% for the child, adolescent, and adult versions respectively.

To the best of our knowledge, only a few studies have applied ML as screening tools for autism in toddlers. Akter and colleagues (2019) [35] analyzed the Q-CHAT-10, collected using the dataset from the AutismTests app, and found that a range of different classifiers, when optimized, could effectively classify autism with accuracy of 98%. Following this line of evidence, we applied different ML approaches to investigate the accuracy and reliability of the Q−CHAT to classify young children as being autistic or typically developing. We used five different machine-learning (ML) algorithms (random forest (RF), naïve Bayes (NB), support vector machine (SVM), logistic regression (LR), and k-nearest neighbor (KNN)) to analyze the complete set of Q-CHAT items and the best subset of discriminating items in a sample of clinically referred young autistic children compared to typically developing children. Furthermore, we explored the cross-cultural validity of the obtained results with ML in our Italian sample.

## 2. Materials and Methods

### 2.1. Participants

**Figure 1:**
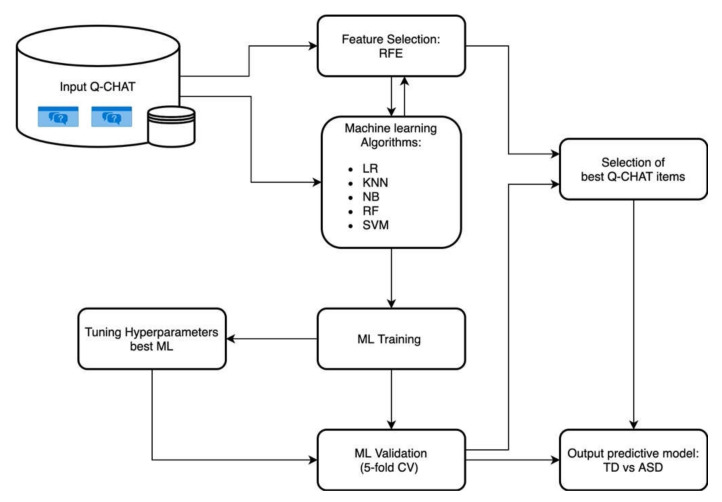
Overview of the entire analytical process. Collected questionnaires processed with machine-learning (ML) models and a feature-selection algorithm. Training phase (ML training) and validation (ML validation) used fivefold cross-validation. Lastly, hyperparameters were automatically tuned on the best-evaluated ML model, and output performance was reported.

In this study, we used a machine-learning approach to analyze a previously collected dataset of young autistic and typically developing children who were administered the Q-CHAT to explore the psychometric characteristics of the instrument in a multicenter study of different Italian regions (Sicily, Tuscany, and Piedmont). For the detailed sociodemographic and clinical characteristics of the sample, refer to Ruta et al. (2019) [14]. A group of n = 126 typically developing (TD) children (mean age (SD) = 33.2 (9.3) months) and n = 139 autistic children (mean age (SD) = 31.6 (8.0) months) were included in the analysis. The main contribution of this work is summarized in Figure 1.

### 2.2. Data Selection and Machine-Learning Classifier Tuning

The classifier was constructed using the Italian Q-CHAT data repository of n = 265 children with an age range between 22 and 43 months. Any individual with more than 25% of missing answers was excluded from analysis. n = 6 children were excluded for this reason, and the final dataset included n = 137 subjects with autism and n = 122 TD children. The processed information was based on the Q-CHAT questionnaire, consisting of 25 items related to the child’s development reflecting autistic traits. Each item (representing a feature for our dataset) was rated on a five-point Likert scale (0–4), with higher ratings indicating more autistic traits, and a Q-CHAT total score ranging from 0 to 100. We applied a supervised approach of binary classification, dividing the dataset into two classes according to diagnostic category (autism vs TD). We tested the five most representative supervised classifiers to understand the intrinsic relationship between Q-CHAT items and the diagnostic label: random forest (RF) [36], naïve Bayes (NB) [37], support vector machine (SVM) [38], K-nearest neighbors (KNN) [39], and logistic-regression (LR) algorithms [40]. Once we found the best-performing predictive model, we conducted parameter tuning on it, applying a two-level grid search (GS) [41] to set the optimal hyper-parameters. Classification of autism vs TD was carried out performing fivefold cross-validation to test the accuracy of each classifier [42]. We trained the ML algorithms on a laptop equipped with an i7-8550U, 8 GB RAM, 256 GB SSD processor, using an Ubuntu 18.04.4 LTS operating system. We used the pandas and NumPy libraries for data manipulation, and the scikit-learn package v.0.22.1 [43] for machine learning in Python.

### 2.3. Feature Selection

Once ML classification with all 25 Q-CHAT items was completed, we selected a subset of features in order to identify a faster screening tool without compromising the screening accuracy and reliability of the Q-CHAT. The support vector machine–recursive feature elimination (SVM–RFE) algorithm with fivefold cross-validation was applied. We selected this method since it is considered one of the most powerful tools to analyze datasets in real scenarios that have few observations and a large number of predictors, or to build predictive models by assessing the importance of the predictor variables [44]. RFE is a recursive process that ranks and selects features according to some score function with the highest score. In particular, it uses a supervised learning estimator that finds the importance of each feature by pruning from the current set of features and recursively repeating it until the best number of features to select is reached. In our experiments, we used SVM with a linear kernel as estimator by running it after each RFE iteration to assess all possible subsets of attributes. This process was repeated until the highest classification accuracy was obtained [45].

### 2.4. Metric for ML Performance

A receiver operating characteristic (ROC) curve of each ML accuracy measurement was produced to plot the sensitivity and 1-specificity of testing set in relation to both autism and TD diagnosis. Area under the curve (AUC) is a measure of the overall predictive validity, where AUC = 0.50 indicates a random prediction of the independent variable, and AUC > 0.90 indicates excellent validity.

The most common metrics for binary classification models are based on standard definitions, namely, true positive (TP), true negative (TN), false positive (FP), and false negative (FN), which represent the number of instances truly classified (TP, TN) and misclassified (FP, FN). From these parameters, a number of model performance metrics can be derived. The most common metric is accuracy, which represents the overall success rate of each classifier and is computed as: accuracy = (TP + TN)/(TP + FP + FN + TN). Other performance metrics include sensitivity/recall, defined as the percentage of correctly classified instances, and is computed as: sensitivity/recall = TP/(TP + FN), and specificity/positive predictive value (PPV), defined as percentage of incorrectly classified instances and computed as: specificity/PPV = TP/(TP + FP). F1 score computed as F1 = (2TP)/(2TP + FP + FN) is the measure of a test’s accuracy and it is based on the harmonic mean of specificity and sensitivity. It reaches its best value at 1 and worst at 0. Lastly, the different types of ML fivefold accuracy were compared using the nonparametric Friedman one-way repeated measure analysis. When the results of Friedman test were statistically significant, comparisons of groups were achieved using the post hoc nonparametric Wilcoxon rank-sum test.

## 3. Results

**Figure 2:**
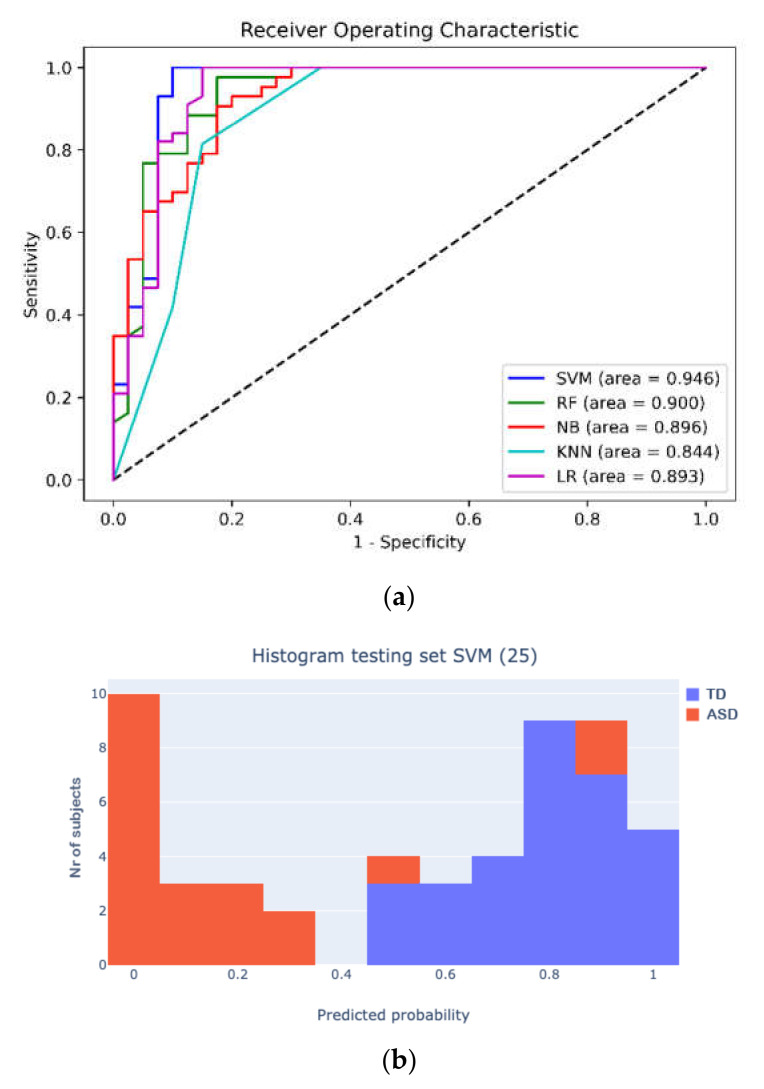
(**a**) Area under curve for Quantitative CHecklist for Autism in Toddlers (Q-CHAT; autism vs typically developing (TD)) comparing all 5 machine-learning models with all features; (**b**) histogram of predictions of best-performing model (SVM).

All five ML algorithms achieved overall accuracy of 90%, with the SVM showing the best discriminant validity. The area under the curve in ROC analysis, reported in Figure 2a, confirmed the higher performance of the SVM (95%) with respect to the RF (90%), NB (89%), LR (89%), and KNN (84%) using all features. Furthermore, Figure 2b shows the histogram of the testing set with the results of the best-performing SVM (25).

Table 1 shows the best hyperparameter configuration of SVM with 14 features after two tuning-level GS.

**Figure 3:**
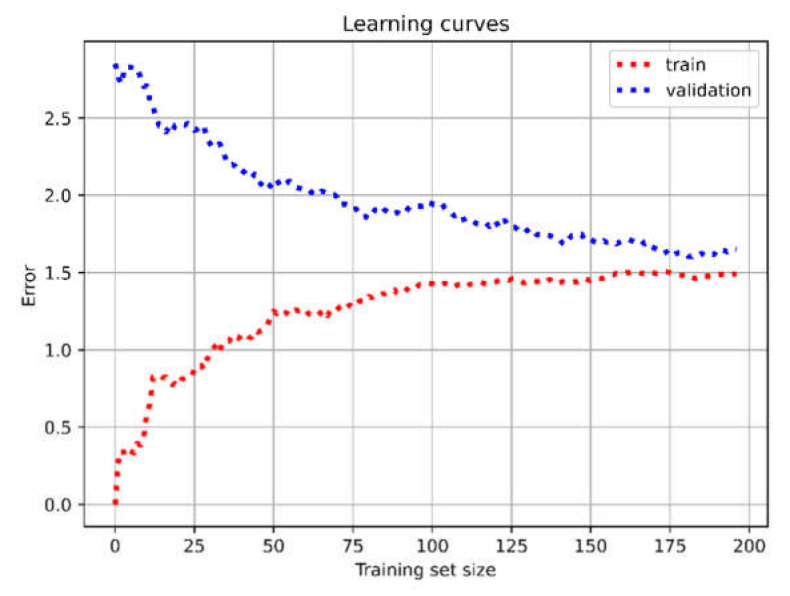
Learning curves to diagnose SVM model performance.

Moreover, for SVM, we used learning-curve analysis [46] to examine the level of classification and monitor the presence of under-/overfitting problems. In our case, Figure 3 shows that, by increasing the training-set size, both training and validation curves converged to a very good reduction in error values, proving that the model did not need more data for further training, and tuning was in line with the trends of a good classifier.

**Figure 4:**
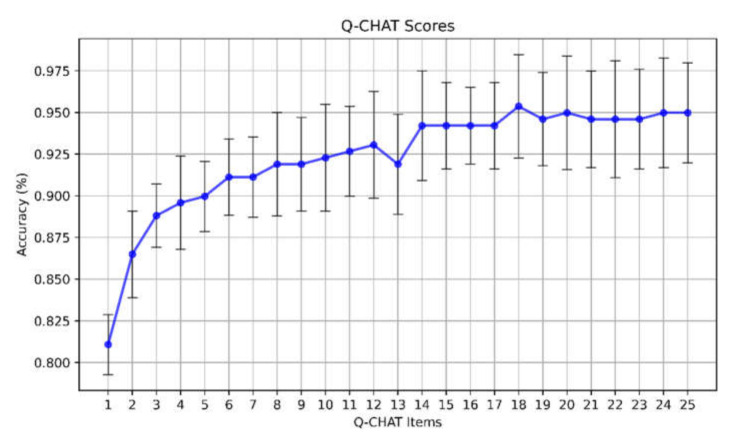
Accuracy selecting an increasing number of Q-CHAT items using SVM–recursive feature elimination (RFE) algorithm.

When we applied the SVM–RFE algorithm with fivefold cross-validation to select the best pool of discriminant items and reduce the computational cost of modeling, performance improved as the number of features increased and peaked around 14, reaching mean accuracy of about 93% (Figure 4).

The 14 selected items, ordered by accuracy using an integrated rank scoring, are: q01, q02, q19, q04, q05, q06, q07, q09, q16, q17, q03, q25, q18, q22. As shown in Table 2, eight items (q01, q02, q19, q05, q06, q09, q17, q25 matched with the same color) were in common with those reported by Allison and colleagues in a previous study where the Q-CHAT-10 was composed of the 10 best discriminating items [20].

To explore the replicability of the Q-CHAT-10 results in our sample, we also ran the five ML algorithms on the 10 items selected by Allison and colleagues, and on the 3 most discriminating items, which were in common between and Allison’s and our study. Table 3 reports the performance of the five classifiers using fivefold cross-validation in relation to the original 25-item Q-CHAT, the 14 items selected by the SVM-RFE algorithm, the 10 items selected by Allison and colleagues, and the 3 most discriminating items in common in the two studies. The SVM algorithm confirmed overall the best accuracy for each Q-CHAT version (25, 14, 10, and 3 items). We extracted the positive predictive value (PPV), sensitivity, and F1 score for each class (autism vs TD) from the 52 participants of the test set, to cross-examine the validity of the trained ML models. The Friedman test showed that there was no significant difference in ML accuracy (*p* > 0.05). The only difference was observed for SVM (25) (X2(4) = 14.86, *p* = 0.005) which had significantly higher accuracy values than those of RF (25), NB (25), kNN (25) and LR (25) (Wilcoxon rank-sum analysis: *p* = 0.031; *p* = 0.016; *p* = 0.008; *p* = 0.016, respectively).

## 4. Discussion

In this study, we applied machine learning and computational intelligence to improve the classification accuracy of the Q-CHAT and to investigate the best subset of items that can efficiently discriminate between young autistic and typically developing children. We tested five different machine-learning classifiers (SVM, RF, NB, LR, KNN). The top-performing SVM model reached overall accuracy of 95% with sensitivity and specificity of 90% and 100%, respectively, compared to RF (sensitivity = 85% and specificity = 95%), NB (sensitivity = 82% and specificity = 100%), LR (sensitivity = 89% and specificity = 91%), and KNN (sensitivity = 81% and specificity = 96%). If we compare these results with those obtained applying standard ROC analysis to the same participant sample [14], we found that ML algorithms were able to improve the classification accuracy of the Q-CHAT. In the previous study [14], the ROC curve showed accuracy of 89.5% (vs. 95%), sensitivity = 83% (vs. 90%), and specificity = 78% (vs. 100%). Furthermore, by running the SVM-RF algorithm, we selected a subgroup of 14 items that maintained very high accuracy, sensitivity, and specificity (91%, 87%, and 96%, respectively, for SVM). In our sample, 8 out of the 14 items (q01, q02, q019, q5, q06, q9, q17, q05) were in common with the Q-CHAT-10 by Allison and colleagues (2012) [20]. To further explore the cross-cultural validity of the instrument, we applied the five ML classifiers to the 10 items selected by Allison and colleagues on the Q-CHAT-10 (2012) [20]. In our sample, the SVM algorithm was able to classify autism with 87% accuracy, 65% sensitivity, and 86% specificity. These results are also in line with those recently reported by Akter et al. (2019) [35]. In their study, Akter and colleagues analyzed a dataset of Q-CHAT-10 administered using a mobile application [33] using ML, and the SVM algorithm was able to classify autism with 98% accuracy. Taking these findings together, our study confirmed the satisfactory cross-cultural validity of the Q-CHAT in different samples, countries, and languages. Furthermore, we looked at the specific items in common between the Q-CHAT-10 and our subset of items, and the three items with the highest ranking in our analysis (q01, q02, q019) were the same as those with the highest PPVs in Allison’s study [20]. Just these 3 items in our SVM algorithm were able to classify autism with accuracy of 83%, sensitivity of 78%, and specificity of 93%. These items refer to reduced response to name, eye contact, and use of gestures, which strongly tap into core autism symptoms related to social orienting and communication, and have been consistently picked up as reliable early markers for autism (see the NICE [47] and the CDC [48] guidelines). Convergent evidence shows that, “unusual eye contact” is 1 of 8 selected ADOS items able to classify autism with nearly 100% sensitivity and 94% specificity [21]. “Direct gaze” on the ADI-R was one of the 3 most discriminant items using a novel ML fusion approach in another study by Bone and colleagues (2016) [29]. We used fivefold cross-validation to improve the estimation of the performance of ML models. Furthermore, we provided a well-balanced dataset between autism and TD children (139 autism vs 126 TD) to train the ML models, which helped us to work with the real dataset, avoiding strategies such as repeated random undersampling that may miss important samples by chance and create bias in terms of the assessment of metrics for ML performance. Nevertheless, further validation studies with a larger cohort are needed, and efforts are currently underway to improve the performance of our models and pave the way to test other sophisticated ML algorithms that require more data.

Overall, the study provides relevant results and implications for autism research and clinical practice. Our study shows that just 3 Q-CHAT items are able to predict an autism condition with high accuracy, closely mapping into previously reported early autism markers. The translation of such quick and reliable models into clinical practice may significantly support large-scale early screening, and timely access to early diagnosis and treatment with evidence-based intervention, with substantial impact on developmental trajectories and prognosis. The dimensional structure of the Q-CHAT, and the high concordance and replicability in cross-cultural settings put the questionnaire in a good position to be used by the pediatric population. However, before translating the use of the screener into clinical practice, additional steps are required, such as determining how features are measured (in what context, with what media, by which practitioners). These aspects are equally important to ensure scaling without loss of accuracy. 

## 5. Conclusions

In this study we investigated the performance of five well-known classification algorithms—RF, NB, SVM, LR, and KNN—to correctly detect autism using items from a quantitative screening tool for autism likelihood in toddlers, namely, the Q-CHAT. Our results show that ML classifiers can, with very high accuracy, classify autistic versus TD children with a small subset of Q-CHAT items. In particular, ML algorithms were able to correctly detect autism with accuracy above 90% from a selection of 14 items, and above 80% using only 3 items. Furthermore, these 3 items were the best discriminating items already selected in the short-form Q-CHAT-10. Taken together, these findings confirm the cross-cultural validity of the Q-CHAT as an early quantitative screening tool for autism, and the potential use of ML to improve accuracy. Further validation studies in large-scale independent samples with different ML models and settings are warranted before translating into clinical practice in primary care. Nevertheless, our findings demonstrate that ML was able to dramatically reduce the number of items, which was sufficient to accurately predict an autism condition, with important implications for facilitating more effective and quick screening procedures, and reaching a significantly greater proportion of the population who are likely to be autistic.

## Figures and Tables

**Table 1:** Tuning support vector machine (SVM) hyperparameters values via grid search.

Model (No. of Selected Features)	Parameters	Kernel	Min	Max	Steps	Scale
**First Hyperparameter-Tuning Level**						
SVM(14)	C	linear, poly, rbf, sigmoid	1	1000	10	logarithmic
	degree	poly	2	9	1	linear
	γ	poly, rbf, sigmoid	0.001	1	10	logarithmic
**Second Hyperparameter-Tuning Level**						
SVM(14)	C	rbf	0.2	4	0.2	linear
	γ	rbf	0.02	0.3	0.02	linear

**Table 2:** Comparison between 14 most discriminating Q-CHAT items (ordered by rank) from SVM–RFE algorithm and 10 most predicting items (ordered by PPV) identified by Allison et al. (2012). Colors highlight eight common items.

Q-CHAT 14 Items (Ordered by Rank SVM–RFE)	Q-CHAT 10 Items (Allison Et Al.; Ordered By PPV)
Does your child look at you when you call their name? (1)	Does your child look at you when you call their name? (1)
How easy is it for you to have eye contact with your child? (2)	How easy is it for you to have eye contact with your child? (2)
Does your child use simple gestures (e.g., wave goodbye)? (19)	Does your child use simple gestures (e.g., wave goodbye)? (19)
Can other people easily understand your child’s speech? (4)	Would you describe your child’s first words as typical? (17)
Does your child point to indicate that they want something (e.g., a toy that is out of reach) (5)	Does your child point to indicate that they want something (e.g., a toy that is out of reach)? (5)
Does your child point to share interest with you (e.g., pointing at an interesting sight)? (6)	Does your child point to share interest with you (e.g., pointing at an interesting sight)? (6)
How long can your child’s interest be maintained by a spinning object (e.g., washing machine, electric fan, toy car wheels)? (7)	Does your child follow where you are looking? (10)
Does your child pretend (e.g., care for dolls, talk on a toy phone)? (9)	Does your child pretend (e.g., care for dolls, talk on a toy phone)? (9)
Does your child do the same thing over and over again (e.g., running the tap, turning the light switch on and off, opening and closing doors)? (16)	Does your child stare at nothing with no apparent purpose? (25)
Would you describe your child’s first words as typical? (17)	If you or someone else in the family is visibly upset, does your child show signs of wanting to comfort them? (e.g., stroking their hair, hugging them)? (15)
When your child is playing alone, do they line objects up? (3)	
Does your child stare at nothing with no apparent purpose? (25)	
Does your child echo things they hear (e.g., things that you say, lines from songs or movies, sounds)? (18)	
How long can your child’s interest be maintained by just one or two objects? (22)	

**Table 3:** Detailed performance metrics of five selected machine-learning classifiers (SVM, random forest (RF), naïve Bayes (NB), logistic regression (LR), k-nearest neighbor (KNN)) with fivefold cross-validation with respect to the original 25-item Q-CHAT, 14 items selected by the SVM–RFE algorithm, 10 items selected by Allison and colleagues, and the 3 most discriminating items in common in the two studies. Note: ASD, autism spectrum disorder.

Model (No. of Selected Features)	Accuracy	Classes	PPV	Sensitivity	F1 Score	No. of Subjects for Clinical Validation
SVM (25)	0.95 (± 0.02)	ASD	1.00	0.90 (±0.04)	0.95 (±0.03)	24
		TD	0.91 (±0.04)	1.00	0.96 (±0.03)	28
SVM (14)	0.91 (± 0.02)	ASD	0.95 (±0.03)	0.86 (±0.01)	0.90 (±0.02)	24
		TD	0.86 (±0.01)	0.95 (±0.03)	0.90 (±0.02)	28
SVM (10; Allison et al.)	0.87 (± 0.03)	ASD	0.79 (±0.03)	0.65 (±0.04)	0.71 (±0.03)	24
		TD	0.76 (±0.04)	0.86 (±0.03)	0.81 (±0.03)	28
SVM (3)	0.83 (± 0.05)	ASD	0.90 (±0.07)	0.78 (±0.07)	0.84 (±0.04)	24
		TD	0.84 (±0.06)	0.93 (±0.07)	0.89 (±0.06)	28
RF (25)	0.90 (± 0.06)	ASD	0.89 (±0.05)	0.74 (±0.08)	0.81 (±0.07)	24
		TD	0.82 (±0.07)	0.93 (±0.06)	0.87 (±0.06)	28
RF (14)	0.88 (± 0.04)	ASD	0.86 (±0.04)	0.83 (±0.06)	0.84 (±0.05)	24
		TD	0.87 (±0.05)	0.90 (±0.04)	0.88 (±0.04)	28
RF (10; Allison et al.)	0.84 (± 0.03)	ASD	0.89 (±0.07)	0.70 (±0.06)	0.78 (±0.06)	24
		TD	0.79 (±0.05)	0.93 (±0.06)	0.86 (±0.06)	28
RF (3)	0.83 (± 0.05)	ASD	0.83 (±0.06)	0.83 (±0.05)	0.83 (±0.06)	24
		TD	0.86 (±0.05)	0.86 (±0.06)	0.86 (±0.06)	28
NB (25)	0.89 (± 0.04)	ASD	1.00	0.70 (±0.02)	0.82 (±0.01)	24
		TD	0.81 (±0.02)	1.00	0.89 (±0.01)	28
NB (14)	0.88 (± 0.04)	ASD	1.00	0.74 (±0.02)	0.85 (±0.01)	24
		TD	0.83 (±0.02)	1.00	0.91 (±0.01)	28
NB (10; Allison et al.)	0.82 (± 0.03)	ASD	1.00	0.65 (±0.02)	0.79 (±0.01)	24
		TD	0.78 (±0.02)	1.00	0.88 (±0.01)	28
NB (3)	0.84 (± 0.03)	ASD	0.82 (±0.07)	0.78 (±0.05)	0.80 (±0.06)	24
		TD	0.83 (±0.06)	0.86 (±0.06)	0.85 (±0.06)	28
KNN(25)	0.83 (± 0.03)	ASD	0.95 (±0.03)	0.71 (±0.05)	0.81 (±0.03)	24
		TD	0.75 (±0.03)	0.96 (±0.02)	0.84 (±0.02)	28
KNN(14)	0.85 (± 0.04)	ASD	0.98 (±0.02)	0.73 (±0.08)	0.84 (±0.05)	24
		TD	0.77 (±0.05)	0.98 (±0.02)	0.86 (±0.03)	28
KNN(10; Allison et al.)	0.83 (± 0.03)	ASD	0.90 (±0.05)	0.76 (±0.04)	0.83 (±0.03)	24
		TD	0.77 (±0.04)	0.91 (±0.05)	0.83 (±0.03)	28
KNN (3)	0.66 (± 0.05)	ASD	0.62 (±0.03)	0.90 (±0.05)	0.73 (±0.03)	24
		TD	0.77 (±0.03)	0.39 (±0.07)	0.52 (±0.08)	28
LR(25)	0.89 (± 0.03)	ASD	0.92 (±0.05)	0.87 (±0.05)	0.89 (±0.03)	24
		TD	0.87 (±0.05)	0.91 (±0.06)	0.88 (±0.03)	28
LR (14)	0.90 (± 0.02)	ASD	0.93 (±0.03)	0.87 (±0.03)	0.90 (±0.02)	24
		TD	0.87 (±0.03)	0.93 (±0.03)	0.90 (±0.02)	28
LR (10; Allison et al.)	0.88 (± 0.03)	ASD	0.92 (±0.04)	0.84 (±0.06)	0.88 (±0.03)	24
		TD	0.84 (±0.05)	0.91 (±0.05)	0.87 (±0.03)	28
LR (3)	0.84 (± 0.05)	ASD	0.84 (±0.05)	0.87 (±0.06)	0.85 (±0.04)	24
		TD	0.84 (±0.06)	0.81 (±0.08)	0.82 (±0.06)	28

## Data Availability

The informed-consent forms signed by the subjects prevent data from being publicly available. Data may be requested via email by researchers, upon reasonable request and verification of all ethical aspects, at gennaro.tartarisco@cnr.it.
